# Supplementary figures and images for: Streptococcus pneumoniae serotype 22F infection in respiratory syncytial virus infected neonatal lambs enhances morbidity
Source: PLoS One. 2021 Mar 11;16(3):e0235026. doi: 10.1371/journal.pone.0235026 (PMC7951856; doi:10.1371/journal.pone.0235026)

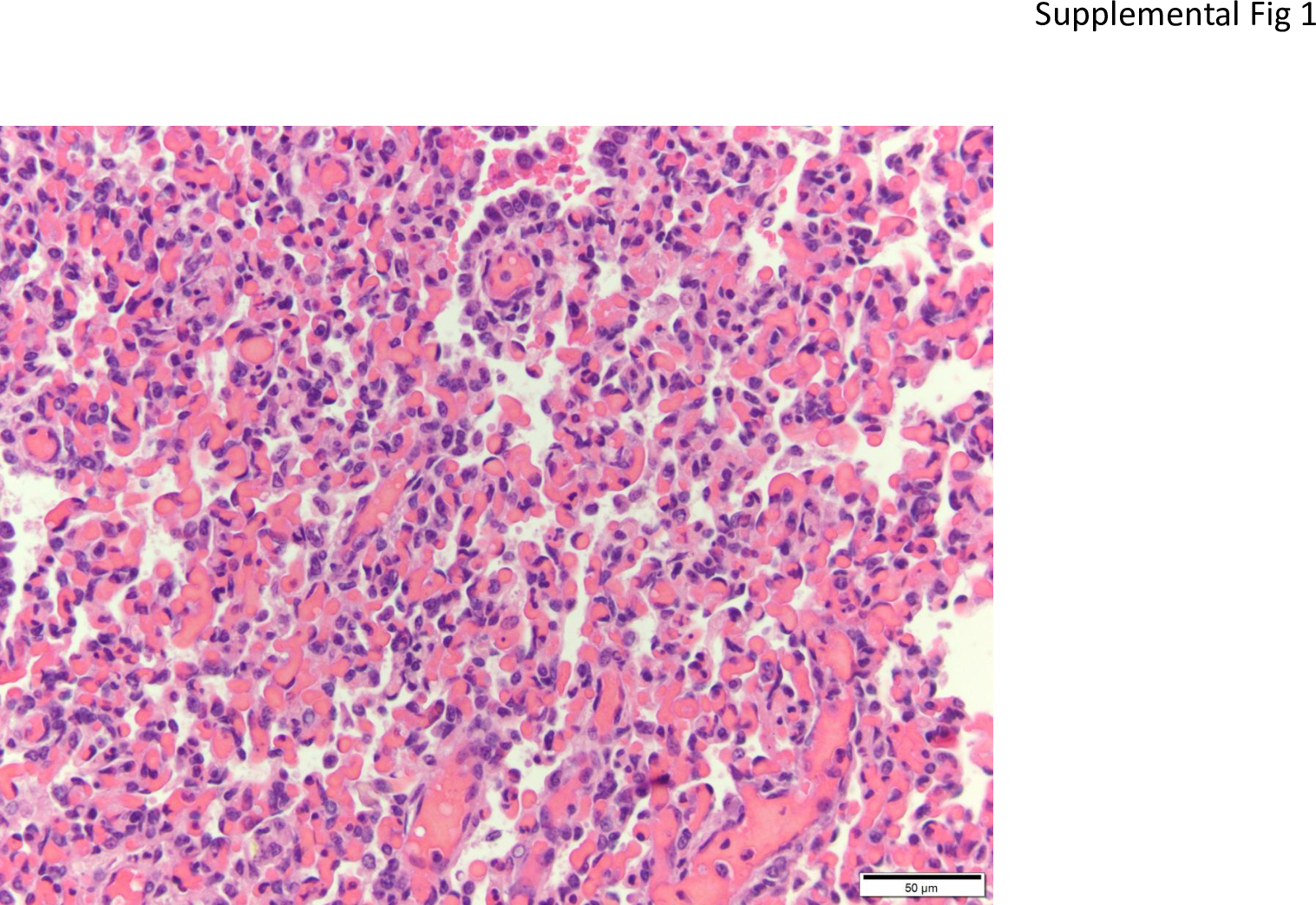

Supplement: S1 Fig — Representative photo of this pathology from Spn only group. This was seen mostly in the Spn -only infected group. (TIF) [file pone.0235026.s001.tif]

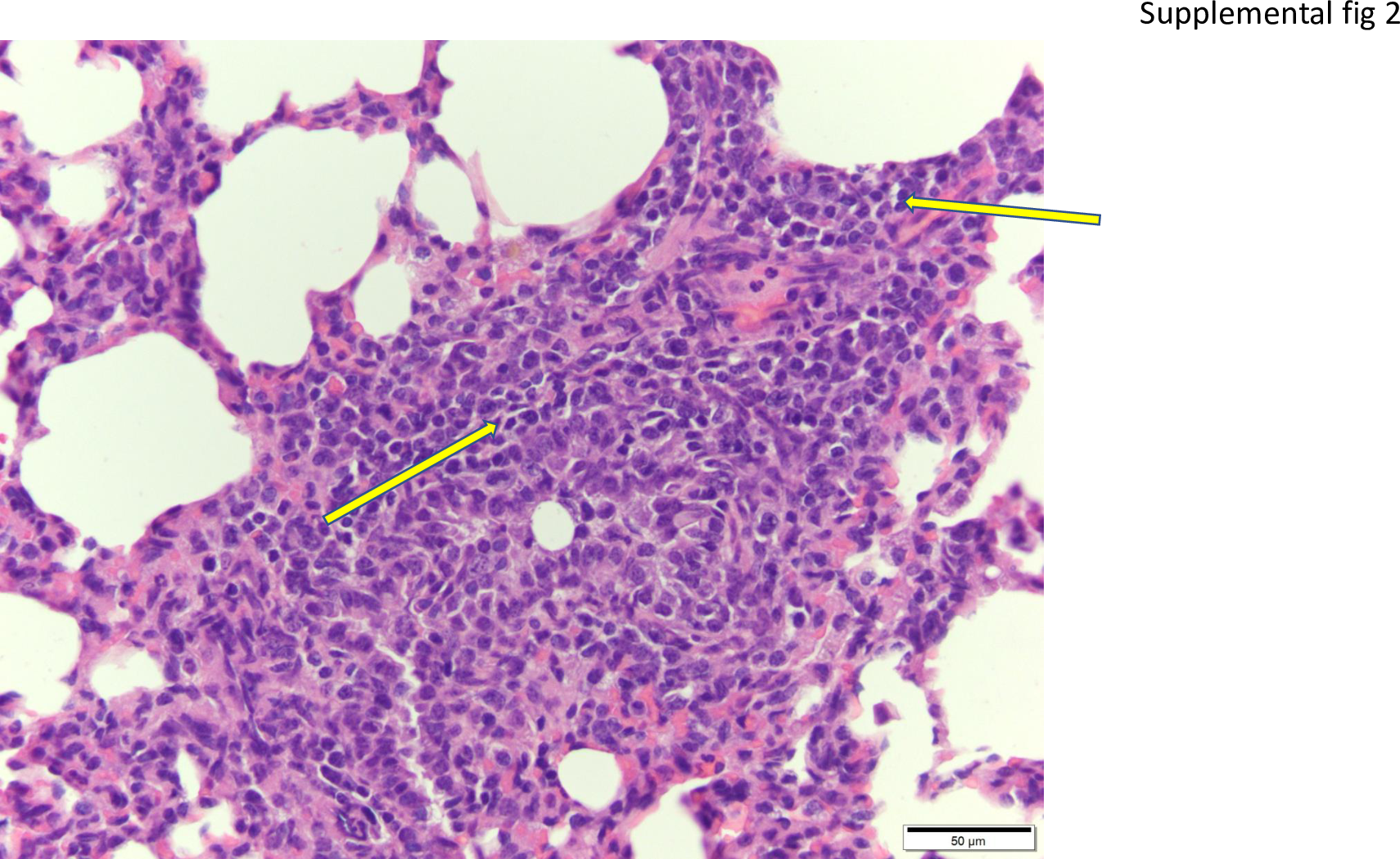

Supplement: S2 Fig — Representative photo of this pathology from RSV only group. (TIF) [file pone.0235026.s002.tif]

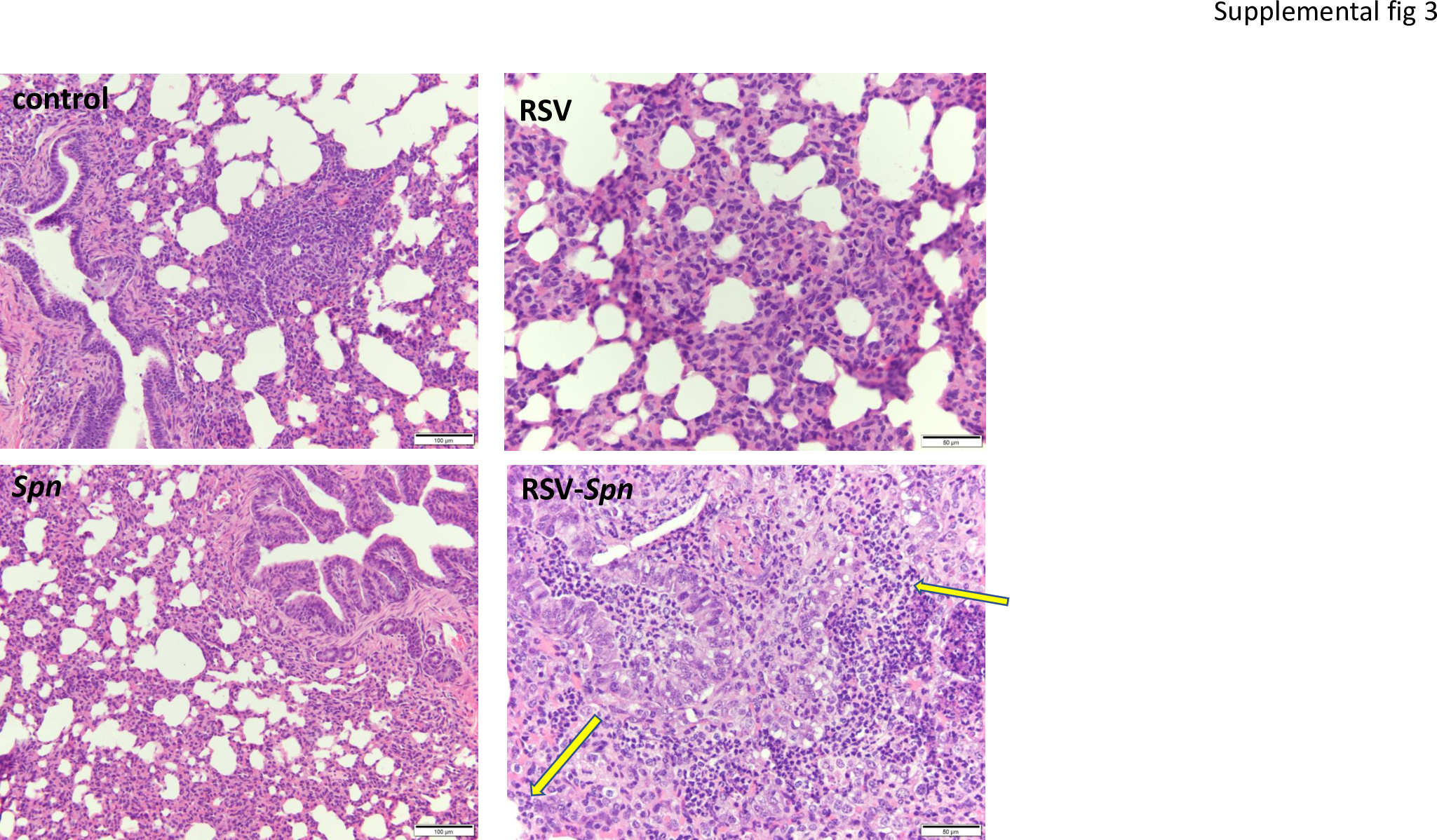

Supplement: S3 Fig — Representative photos of each group looking for neutrophils where large clusters of neutrophils (yellow arrows pointing to neutrophil clusters) were only found in the dual infection group. (TIF) [file pone.0235026.s003.tif]
